# Gender diversity and autism spectrum disorder in child and youth population

**DOI:** 10.1192/j.eurpsy.2024.963

**Published:** 2024-08-27

**Authors:** P. R. Rodríguez, M. M. Grimal, E. E. M. Castellano, F. A. R. Batista, N. M. Pérez

**Affiliations:** Psychiatry, Hospital Universitario de Gran Canaria Doctor Negrín, Las Palmas de Gran Canaria, Spain

## Abstract

**Introduction:**

Interest in the co-occurrence of gender dysphoria and autism spectrum disorder has gained prominence in recent years. Gender dysphoria refers to the distress experienced when there is an incongruence between gender identity and sex assigned at birth. On the other hand, autism spectrum disorder is characterized by difficulties in communication and social interaction, as well as restrictive and repetitive patterns of behavior.

**Objectives:**

The aim of this paper is to review the current available literature in order to expand our knowledge about gender identity and dysphoria in the population with autism spectrum disorder.

**Methods:**

A qualitative review was conducted over the last 20 years, using the Medline database through PubMed. Combinations of MeSH terms related to gender identity and people with autism spectrum disorder were used, selecting those studies in English, French or Spanish that met the objectives of the review, excluding references in other languages. The scientific evidence obtained was analyzed and synthesized.

**Results:**

The development of gender identity of people with autism spectrum disorder can be a complex process. Comparing the general population with the population with autism spectrum disorder, a higher prevalence of gender dysphoria has been evidenced in the population with autism spectrum disorder, and within this group when segmented by gender, greater in women than in men.

**Conclusions:**

This review highlights the importance of increasing knowledge about sexuality and gender dysphoria in people with autism spectrum disorder in order to facilitate the development, understanding and acceptance of their gender identity and sexual orientation of these people.

**Disclosure of Interest:**

None Declared

